# Variant spectrum of *F8* and *F9* in hemophilia patients from southern China and 26 novel variants

**DOI:** 10.3389/fgene.2023.1254265

**Published:** 2023-12-22

**Authors:** Fucheng Li, Liya He, Guilan Chen, Yan Lu, Ru Li, Yongling Zhang, Xiangyi Jing, Rujuan Ling, Dongzhi Li, Can Liao

**Affiliations:** ^1^ Department of Prenatal Diagnostic Center, Guangzhou Women and Children’s Medical Center, Guangzhou Medical University, Guangzhou, Guangdong, China; ^2^ Hematology Department, Guangzhou Women and Children’s Medical Center, Guangzhou Medical University, Guangzhou, Guangdong, China; ^3^ Department of Internal Medicine, Guangzhou Women and Children’s Medical Center, Guangzhou Medical University, Guangzhou, Guangdong, China

**Keywords:** hemophilia, F8, F9, variant, prenatal diagnosis

## Abstract

Hemophilia, an X-linked recessive disorder, is characterized by spontaneous or trauma-induced prolonged bleeding. It is classified as hemophilia A when caused by variants in the *F8* gene, and hemophilia B when caused by *F9* variants. Few studies have described hemophilia variants in the Chinese population. This study aimed to investigate the clinical and genetic profiles of 193 hemophilia patients from southern China. Utilizing Sanger sequencing, multiplex ligation-dependent probe amplification, gap detection, long-range PCR, and multiplex PCR, we identified both *F8* and *F9* gene variants. Pregnant women with a history of hemophilia A offspring underwent amniocentesis or villus sampling for the variant detection. Variants in *F8* and *F9* were pinpointed in 183 patients, with 26 being novel discoveries. Notably, genetic testing was absent in the initial evaluation of 133 out of 161 patients, leading to a protracted average definitive diagnosis timeline of 2 years. Remarkably, two hemophilia A cases with anticipated severe phenotypes due to protein-truncating variants presented with only moderate or mild clinical manifestations. Among the 40 fetuses tested, 34 were males, with 17 exhibiting hemizygous variants in the *F8* gene. Our results contribute to the broader understanding of *F8* and *F9* variant spectrum and highlight the underuse of genetic analyses in southern China.

## Introduction

Hemophilia is a rare X-linked recessive disorder characterized by spontaneous or prolonged bleeding from various tissues and organs. Common bleeding sites include the joints, nose, and brain (Fogarty et al., 2013; Josephson, 2013). Hemophilia can be classified as either hemophilia A (HA) (OMIM: 306700), caused by variants of *F8* (Gene ID: 2157), or hemophilia B (HB) (OMIM: 306900), caused by variants of *F9* (Gene ID: 2158). The pathogenic variants of *F8* and *F*9 may result in the deficiency of two crucial components of the clotting cascade: coagulation factor VIII (FVIII) and factor IX (FIX), respectively. Distinguishing between HA and HB based on clinical features alone is challenging (Castaman and Matino, 2019), despite the accepted notion that HB is less severe than HA (Mannucci and Franchini, 2013). HA and HB can be diagnosed by measuring plasma factor VIII clotting activity (FVIII:C) and factor IX clotting activity (FIX:C) respectively, along with normal and functional von Willebrand factor levels. Depending on FVIII:C and FIX:C levels, hemophilia can be classified as severe (<1%), moderate (1%–5%), or mild (>5–40%) (Bolton-Maggs and Pasi, 2003). It is important to note that HB may be misdiagnosed in infants under 6 months of age because of their naturally low FIX:C levels (Andrew et al., 1987). Genetic analysis plays a crucial role in confirming the diagnoses of HA and HB by enabling the identification of hemizygous pathogenic *F8* and *F9* variants in males respectively. Heterozygous women may also be affected by hemophilia due to skewing inactivation of the X chromosome (Plug et al., 2006). A rare condition is observed in women harboring compound heterozygous or homozygous pathogenic variants. Hemophilia treatment typically involves the intravenous infusion of FVIII or FIX concentrate. However, a significant replacement therapy complication is the development of inhibitory antibodies against the therapeutic exogenous factor (Oldenburg and Pavlova, 2006; Gouw et al., 2012; Eckhardt et al., 2013). Approximately 25%–30% of patients with severe HA (Knobe et al., 2000) and 5%–10% of patients with severe HB (Male et al., 2021; Johnsen et al., 2022) develop such inhibitors, with variations influenced by ethnicity and variant type (Aledort and Dimichele, 1998; Carpenter et al., 2012). The likelihood of inhibitor formation is notably higher in patients with large deletions, nonsense variants, small deletions and insertions (indels), and splice site variants (Rosendaal et al., 2017). For both HA and HB patients, the formation of inhibitors is predominantly associated with disruptive structural variants such as large deletions. Conversely, missense variants exhibit a lower incidence of inhibitor development (Oldenburg and Pavlova, 2006; Gouw et al., 2012; Rallapalli et al., 2013). It is worth noting that the nonsense variants occurring in the B domain of *F8* are associated with the lower inhibitor because the premature stop codon caused by nonsense variants in the B domain may undergo translational readthrough, resulting in the maintenance of the A3-C2 domain. The existence of the A3-C2 domain has been shown to lessen the immunogenicity of therapeutic FVIII and then decrease the formation of inhibitors (Testa et al., 2023). Prenatal testing is crucial for the mothers of affected patients in order to prevent the recurrence of variants in subsequent pregnancies. To date, numerous *F8* and *F9* variants have been reported in different populations, including Chinese cohorts (https://dbs.eahad.org/) (Xue et al., 2010; Guo et al., 2018; Luna-Záizar et al., 2018; Chen et al., 2021). This study aimed to investigate the clinical and genetic features of hemophilia in southern China by recruiting patients with HA and HB and conducting clinical as well as genetic analyses. Our findings are expected to broaden the variant spectrum of *F8* and *F9*, providing valuable data for the management of patients with hemophilia and prenatal diagnosis in affected families.

## Materials and methods

### Subjects

Between May 2013 and November 2022, a total of 296 participants from the Guangzhou Women and Children’s Medical Center were recruited for this study. The participants included 161 unrelated patients clinically diagnosed with HA, along with 99 available mothers, as well as 32 unrelated patients clinically diagnosed with HB, along with four available mothers. All participants were of Han Chinese ethnicity from southern China. A genetic analysis was conducted on all 193 patients. When variants were identified, we performed genetic analysis on mothers to determine their origin. The study was approved by the Ethics Committee of the Guangzhou Women and Children’s Medical Center. Written informed consent was obtained from all participants or their guardians, following the principles outlined in the Declaration of Helsinki.

### Laboratory examinations

Laboratory examinations included the measurement of FVIII:C, FIX:C, and inhibitor levels. Peripheral blood samples were collected from the patients in 3.8% citrate tubes, followed by centrifugation at 2500 *g* for 15 min to obtain platelet-poor plasma. Commercial FVIII- and FIX-deficient plasma (Diagnostica Stago, Asnières-sur-Seine, France) were used to measure FVIII:C and FIX:C levels via standard one-stage clotting assay, which is based on the activated partial thromboplastin time (APTT) test. The reagents for FVIII:C and FIX:C testing, are from Diagnostica Stago (Asnières-sur-Seine, France) and include FVIII or FIX-deficient plasma, calibration plasma, APTT activator reagent (Triniclot aPTT S), calcium, quality control plasma, and dilution buffer. All reagents were prepared according to the manufacturer’s protocol. The coagulometer used for the detection is the STA-R Evolution analyzer (Diagnostica Stago, Asnières-sur-Seine, France). The FVIII:C or FIX:C is provided by comparing the measured APTT of the test plasma with those measured in the standard reference plasma, and is expressed as % of normal. The Bethesda assay was used to determine the inhibitor titers (Duncan et al., 2013), it is a method used to evaluate the capacity of test plasma to inactivate the FVIII or FIX. The test plasma is mixed with an equal volume of a normal plasma pool, the FVIII:C or FIX:C of the mixture is measured and then compared with that of the control plasma. The percentage of residual FVIII:C or FIX:C is calculated, and one Bethesda unit (BU) is defined as the amount of inhibitor that results in 50% residual FVIII:C or FIX:C. The BU/mL in the sample is determined from the theoretical inhibitor graph by interpolating the percentage residual activity against Bethesda units.

### Genetic analysis

Genomic DNA was extracted from the peripheral blood of participants using a QIAamp Blood DNA Kit (QIAGEN, Hilden, Germany) and subjected to genetic analysis. A long-range polymerase chain reaction (PCR) was performed to detect the intron 22 (IVS 22) inversion in *F8* according to the manufacturer’s instructions (Yaneng Bioscience, Shenzhen, China). Multiplex PCR was used to detect the intron 1 (IVS 1) inversion in *F8*. Primers were designed using Oligo6.0 (http://www.oligo.net/downloads.html) in order to amplify all exons and exon-intron boundaries of *F8* and *F9* via PCR. The PCR products were then sequenced on an ABI 3730XL Automated DNA Sequencer using the BigDye Terminator v3.1 Cycle Sequencing Kit (Applied Biosystems, Foster City, CA, USA). The GenBank sequences NM_000132.3 for *F8* and NM_000133.3 for *F9* were retrieved from the UCSC database (http://genome.ucsc.edu/) and were used for comparison of our sequencing results for variant detection. The reference sequences for the encoded proteins of *F8* and *F9* were NP_000123.1 and NP_000124.1 respectively. Variant nomenclature was adopted, with +1 corresponding to A of the AUG translation initiation codon, according to the Human Genome Variation Society nomenclature (den Dunnen et al., 2016). When PCR failed for certain exon(s), Multiplex Ligation Dependent Probe Amplification (MLPA) was used to detect large deletions of *F8* and *F9*, according to the manufacturer’s instructions for the SALSA MLPA probemix P178-B2 *F8* and P207-D1 *F9* respectively (MRC-Holland, Amsterdam, Netherlands). Since IVS 22 inversion accounts for approximately 40%–50% of severe HA cases, detection of IVS 22 inversion should be the top priority in patients with severe HA. Sequencing for *F8* and detection of IVS 1 inversion were performed sequentially due to the minimum proportion of IVS 1 inversion (1%–5%) (Antonarakis, et al., 1995; Abelleyro, et al., 2020). Forty pregnant women underwent amniocentesis or villus sampling, and quantitative fluorescence-polymerase chain reaction was used to rule out maternal cell contamination. All 40 fetuses were genetically tested for causative variants, regardless of the sex of the fetuses.

### Variant interpretation

The variants were interpreted in accordance with the recommendations of the American College of Medical Genetics (ACMG) (Richards et al., 2015). The framework classifies variants as pathogenic (P), likely pathogenic (LP), variant of uncertain significance (VUS), benign (B), and likely benign (LB). The criteria for classification are based on a level of strength: stand-alone (A), very strong (VS), strong (S), moderate (M), or supporting (PP). Twenty-six criteria can be applied, including 15 pathogenic criteria: 1 very strong (PVS1), 4 strong (PS1-4), 6 moderate (PM1-6), 4 supporting (PP1-4), and 11 benign criteria: 1 stand-alone (BA1), 4 strong (BS1-4), and 6 supporting (BP1-7 except BP6). The applied criteria are then combined to reach a classification according to the scoring rules in the ACMG/AMP recommendations (Richards et al., 2015). The criteria used in this study are described below. PVS1 is applicable when a variant is predicted to result in nonsense medicated decay (NMD). Automatic PVS1 interpretation (AutoPVS1) (https://autopvs1.bgi.com/) was used to predict whether PVS1 is applicable or should be used as reduced strength for the nonsense, frameshifts, ±1 or 2 canonical splice sites, and initiation codons variants. For single or multi-exon deletions that cannot be predicted by AutoPVS1, NMD prediction is based on the premature termination codon not occurring in the 3′ most exon or the 50 bp in the 3′ most penultimate exon (Abou Tayoun et al., 2018). PS4 is applicable when the prevalence of a variant in affected individuals is significantly increased compared to the prevalence in controls. For the rare variants of *F8* and *F9* that do not provide sufficient power to reach statistical significance due to small sample sizes, a modified strength of PS4 is most applicable. The thresholds for “PS4_Supporting”, “PS4_Moderate”, “PS4”, and “PS4_Very Strong” have been specified as 1, 2, 4, and 16, respectively, for previously reported cases (Lee et al., 2018; Mester et al., 2018). We searched the Human Gene Mutation Database (http://www.hgmd.org/) and two European Association for Haemophilia and Allied Disorders (EAHAD) Coagulation Factor Variant Databases (McVey et al., 2020): Factor VIII Gene (F8) Variant Database (https://f8-db.eahad.org/) and Factor IX Gene (F9) Variant Database (https://f9-db.eahad.org/index.php) to determine whether the variants had been reported. When a missense variant is located in a mutational hot spot and/or a critical and well-established functional domain without benign variation, PM1 is applicable. For the missense variants of *F8* located in the B domain, PM1 is not applicable as the B domain is partially spliced off the mature protein. The domain data were obtained from UniProt (https://www.uniprot.org/). If a variant is absent from a large general population, this observation can be considered a moderate piece of evidence for pathogenicity (PM2). The weight of PM2 criteria should be decreased to supporting according to the Sequence Variant Interpretation Working Group, as the absence or rarity is given too much weight after substantial analysis and modeling (ClinGen Sequence Variant Interpretation Recommendation for PM2-Version 1.0). We searched the Genome Aggregation Database (gnomAD) (http://gnomad-sg.org/) to determine whether the variant is absent from the general population. For the missense variants, we used Rare Exome Variant Ensembl Learner (REVEL) (https://sites.google.com/site/revelgenomics/) to predict the possible effects. The thresholds for the levels of evidence for pathogenicity (PP3) were: ≥0.932 for strong, [0.733, 0.932) for moderate, and [0.644, 0.733) for supporting, and for benign, they were: ≤0.003 for BP4-very strong, (0.003, 0.016] for BP4-strong, (0.016, 0.183] for BP4-moderate, and (0.183, 0.290] for BP4 (Pejaver et al., 2022). To avoid overinterpretation of variants, a moderate strength of PP3 was applied even when the scores were ≥0.932. For the noncanonical splice site variant, SpliceAI (https://github.com/Illumina/SpliceAI) was used to predict the splicing effect. PP3 can be applied when the SpliceAI scores are >0.2, and BP7 can be applied when the SpliceAI scores are <0.01. PP4 is applicable when patient’s phenotype or family history is highly specific for a disease with a single genetic etiology, and PP4 is applied in this study when the FVIII:C or FIX:C is below 40%.

## Results and discussion

### Clinical characteristics of HA and HB patients

Of the 161 unrelated patients clinically diagnosed with HA, 117 were classified as having severe, 16 as having moderate, 19 as having mild, and nine as having disease of unknown severity owing to FVIII:C data not being available (Table 1). Of the 32 unrelated patients diagnosed with HB, 20 had severe, seven had moderate, four had mild disease, and one had disease of unknown severity (Table 1). Finally, 153 HA and 30 HB patients were confirmed via genetic testing. Among these, the mean age of disease onset was 1.7 years (ranging from 1 day to 15 years) for HA and 3.4 years (ranging from 5 days to 11.3 years) for HB, with median values of 9 months and 1.4 years, respectively (Table 2). The diagnosis of hemophilia was typically established at a mean age of 4.3 years (ranging from 14 days to 23 years) for HA and 5.1 years (ranging from 19 days to 12 years) for HB (Table 2). The mean time to a definitive diagnosis was 2.1 years for HA and 1.6 years for HB (Table 2). Notably, genetic testing was offered to only 18% of the HA patients (24/132) and 14% of the HB patients (4/29) during their initial hospital visit, reflecting a lack of awareness regarding *F8* and *F9* genetic analysis in many parts of southern China. Most patients underwent genetic testing only after being referred to our institute, the major referral center for pediatric patients in the region. The lack of awareness regarding genetic analyses leads to low rates of genetic testing during initial hospital visits for patients with HA and HB. This may result in a delayed diagnosis and inadequate genetic counseling.

**TABLE 1:** Characteristic of variant in *F8* and *F9* and inhibitor in this cohort.

Variant type	Severe patients	Moderate patients	Mild patients	Patients with unknown severity	Total patients	Inhibitor	Origin	Number of variants	Number of novel variants
	N (%)	N (%)	N (%)	N (%)	N (%)	N (%)	(*De novo*/inherited)	N (%)	
*F8*									
IVS 22 inversion	49 (41.9)	1 (6.25)	2 (10.5)	0 (0)	52 (32.2)	4/34 (11.8)	4/30	-	-
IVS 1 inversion	3 (2.6)	0 (0)	1 (5.3)	0 (0)	4 (2.5)	0/3 (0)	0/4	-	-
Missense^a^	13 (11.1)	12 (75)	11 (57.9)	4 (44.4)	40 (24.8)	0/32 (0)	0/24	36 (43.4)	5
Nonsense	20 (17.1)	1 (6.25)	0	3 (33.3)	24 (14.9)	4/19 (21)	0/15	18 (21.7)	4
Indel	22 (18.8)	1 (6.25)	0 (0)	2 (22.2)	25 (15.6)	3/21 (14.3)	1/18	22 (26.5)	11
Gross deletion	6 (5.1)	0 (0)	0 (0)	0 (0)	6 (3.7)	2/6 (33.3)	0/2	5 (6)	0
Canonical splice site	2 (1.7)	0 (0)	0 (0)	0 (0)	2 (1.3)	1/2 (50)	0/1	2 (2.4)	0
None	2 (1.7)	1 (6.25)	5 (26.3)	0 (0)	8 (5)	0/0 (0)	NA	0	0
Total	117	16	19	9	161	14/117 (11.9)	5/94	83	20
*F9*									
Missense	11 (55)	7 (100)	2 (50)	1 (100)	21 (65.6)	0/17 (0)	0/3	16 (66.7)	3
Nonsense	3 (15)	0 (0)	0 (0)	0	3 (9.4)	1/3 (33.3)	NA	2 (8.3)	0
Indel	1 (5)	0 (0)	0 (0)	0	1 (3)	0/1 (0)	NA	1 (4.2)	1
Gross deletion	2 (10)	0 (0)	0 (0)	0	2 (6.3)	1/2 (50)	NA	2 (8.3)	1
Canonical splice site	1 (5)	0 (0)	0 (0)	0	1(3.1)	0/1(0)	0/0	1(4.2)	0
Noncanonical splice site	1(5)	0 (0)	1 (25)	0	2 (6.3)	0/1 (0)	0/1	2 (8.3)	1
None	1 (5)	0 (0)	1 (25)	0	2 (6.3)	0/0 (0)	NA	0	0
Total	20	7	4	1	32	2/25 (8)	0/4	24	6

^a^ Two variants c.2535C>A (p.Asp845Glu) and c.394G>A (p.Glu132Lys) were excluded as they appeared with other pathogenic variants in the identical patients respectively and both were classified as likely benign. Indel: Small deletion and insertion. IVS 22: Intron 22. IVS 1: Intron 1. N: Number. NA: not available.

**TABLE 2:** Characteristic of the age of hemophilia A and B patients.

Patient	Age at onset (mean/Median)	Age at confirmed diagnosed (mean/Median)	Duration (mean/Median)	Number of patients with immediate genetic testing^a^
Hemophilia A	1 day - 15 years (1.7 years/9 months)	14 days - 23 years (4.3 years/2 years)	13 days - 20 years (2.1 years/6 months)	24/132
Hemophilia B	5 days - 11.3 years (3.4 years/1.4 years)	19 days - 12 years (5.1 years/2.5 years)	14 days - 8 years (1.6 years/1 year)	4/29
Total	1 day - 15 years (2 years/11 months)	14 days - 23 years (5.2 years/2.5 years)	13 days - 20 years (2 years/6 months)	28/161

^a^ Patients with immediate genetic testing are those who can be subject to genetic analysis at their first visit to hospital due to bleeding related symptoms.

Among the 117 patients with HA who underwent inhibitor testing, 14 (11.9%) developed inhibitors, with only one being mildly affected (Table 1). The incidence of inhibitor development varies across regions, with the highest incidence reported in the American population (30%) and the lowest being in East Asia (11%). The incidence rate in this cohort was similar to that of the overall population in East Asia but higher than that in two other Chinese cohorts (Luna-Záizar et al., 2018). In this cohort, the highest incidence of inhibitors was observed in patients with canonical splice site variants (50%, 1/2), although the limited number of patients may have introduced bias. The incidence rates for other variant types were as follows: gross deletion (33.3%, 2/6), nonsense (21%, 4/19), indel (14.2%, 3/21), and IVS 22 inversion (11.8%, 4/34). These rates were generally consistent with prior findings (Luna-Záizar et al., 2018). A comparable rate of inhibitor development was exhibited among patients with nonsense variants within the B domain (20%, 1/5) and the non-B domain (21%, 3/14). This is different from earlier reports that patients with nonsense variants in the B domain had a lower rate of developing inhibitors than those with non-B domain variants (Testa et al., 2023). This may be due to the bias introduced by the limited number of patients. None of the patients with IVS 1 inversion or missense variants developed inhibitors. Further, only two patients with HB harboring gross deletions and nonsense variants (8%, 2/25) developed inhibitors (Table 1).

### Variants in *F8*

#### Variant spectrum

Variants in the *F8* gene were identified in 153 of the 161 patients with HA, resulting in a variant detection rate of 95% (Table 1). In addition to IVS 22 inversion and IVS 1 inversion, a total of 83 distinct variants were identified, including five large deletions (6%, 5/83), 22 indels (26.5%, 22/83), 36 missense variants (43.4%, 36/83), 18 nonsense variants (21.7%, 18/83), and two canonical splice site variants (2.4%, 2/83) (Table 1). Among these variants, 20 had not been previously reported (Table 1).

Analysis of the 99 available mothers revealed that 94 of the 99 variants (95%) were inherited (Table 1), indicating a higher carrier rate than reported for another Chinese cohort (79%) (Lu et al., 2018). The high rate of inherited variants highlights the increased likelihood of variant recurrence in families with HA in southern China. Therefore, it is crucial for mothers and other female family members to undergo genetic testing before planning a pregnancy. The remaining five cases involved *de novo* variants, including four IVS 22 inversion variants and one small deletion (Table 1). In cases of *de novo* variants, prenatal diagnosis must be performed during subsequent pregnancies to determine whether the variants are germline mosaicism rather than *de novo*, as previously reported (Lu et al., 2018).

No detectable variants were noted in eight patients (5%, 8/161), including two severe, one moderate, and five mild cases. However, this does not rule out the diagnosis of HA. It is possible that the variants are located deep within introns or regulatory regions (Castaman et al., 2011; Bach et al., 2016; Chang et al., 2019; Dericquebourg et al., 2020) or that unknown causal genes may be involved, resulting in decreased FVIII:C levels. Further investigation of these patients is ongoing.

A prenatal diagnosis was performed for 40 pregnant women with a family history of *F8* variants. Hemizygotes were identified in 42.5% (17/40) of the cases (Table 3). Following genetic counseling, decisions were made regarding pregnancy termination for the affected fetuses. Interestingly, 85% (34/40) of the fetuses were males, yielding a sex ratio of 0.85 (male fetus numbers/total fetus numbers), a deviation from reported sex ratio of 0.502 of all embryos from conception to birth (Orzack et al., 2015).

**TABLE 3:** Distribution of variants of *F8* in prenatal diagnosis.

Variant type	Male	Female	Total
	N (Positive^#^/negative)	N (Positive^#^/negative)	N (Positive^#^/negative)
IVS 22 inversion	12 (7/5)	2 (1/1)	14 (8/6)
Missense	7 (3/4)	2 (1/1)	9 (4/5)
Nonsense	7 (4/3)	1 (0/1)	8 (4/4)
Indel	7(3/4)	1 (0/1)	8 (4/4)
Canonical splice site	1 (0/1)	0 (0/0)	1 (0/1)
Total	34 (17/17)	6 (2/4)	40 (20/20)

IVS 22: Intron 22. Indel: Small deletion and insertion. N: Number. # means hemizygote in male and heterozygote in female.

#### Inversion

A total of 52 patients (32.2% of all patients and 41.9% of severe patients) had an IVS 22 inversion (Table 1). IVS-1 inversion was found in only four patients, accounting for 2.5% (4/161) of all patients and 2.6% (3/117) of patients with severe disease. These results are comparable to those reported in other populations (Luna-Záizar et al., 2018). Nearly all patients with IVS 22 inversion were classified as severe, except for four cases (HA1-4) (Table 4). Three patients (HA1-3) carried IVS 22 inversion, and one patient carried IVS 1 inversion. In this study, HA1 was a seven-year-old boy who was diagnosed with HA at the age of 2 years, with an FVIII:C level of 10%. After diagnosis, he received two cryoprecipitate injections for treatment. HA2 was a three-year-old boy whose guardian requested genetic testing after being diagnosed with HA, with an FVIII:C level of 6.6% following cerebral infection and bleeding at 2 months of age. FVIII injections were not administered to this patient. HA3 presented with cutaneous ecchymosis at 6 months of age, and FVIII:C testing revealed a level of 2.6%. No further spontaneous bleeding episodes were observed, and treatment was provided only for the injuries. Hospitalization was required because of a sudden oral hemorrhage, and further testing confirmed the presence of inhibitors. HA4, a patient with an IVS 1 inversion, was a 17-year-old boy. His FVIII:C level was 8% at 1 year of age. He occasionally developed petechiae after the trauma, and at present, his knee joint is severely swollen, preventing him from walking. It is generally believed that the presence of IVS 22 inversion and IVS 1 inversion in patients with moderate or mild HA is either due to residual FVIII clotting activity from prior transfusions or inaccurate test methods (Konkle et al., 2000). In these four patients, FVIII:C testing was conducted before exogenous FVIII therapy. Unfortunately, retesting the patient’s peripheral blood using another method was not possible. In addition to the inaccurate measurement of FVIII:C, one possibility is that patients with mild or moderate disease carrying IVS 22 inversion and IVS 1 inversion harbor undetected variants, such as complex rearrangements associated with IVS 22 inversion and IVS 1 inversion, which preserve the *F8* reading frame. However, further investigation is required to confirm this hypothesis.

**TABLE 4:** The moderately or mildly affected patients with inversion of intron 22 and 1 and protein-truncating variants in *F8.*

Patient	Variant	Variant type	FVIII: C	Inhibitor	Clinical manifestation
HA 1	IVS 22 inversion	Inversion	10	NA	Elbow joint swelling
HA 2	IVS 22 inversion	Inversion	6.6	NA	Intracranial hemorrhage
HA 3	IVS 22 inversion	Inversion	2.6	Yes	Gum bleeding, ecchymoses
HA 4	IVS 1 inversion	Inversion	8	NA	Ecchymoses after injuries, knee swelling
HA 5	c.6496C>T (p.Arg2166*)	Nonsense	1.2	No	Eyelids swelling
HA 6	c.5963_5964del (p.Glu1988Glyfs*3)	Small deletion	2	NA	?

FVIII: C: Factor VIII clotting activity; IVS 22: Intron 22. IVS 1: Intron 1. NA: not available.

#### Protein-truncating variants

Forty protein-truncating variants, including 22 indels and 18 nonsense variants, were identified in 49 patients (Table 1). Long poly-A and poly-T runs (at least 6 consecutive adenines or thymine) are hotspots for indels due to slipped mispairing or intragenic recombination, which are mechanisms responsible for indel formation (Krawczak and Cooper, 1991; Bidichandani et al., 1994; Nakaya et al., 2001; Bogdanova et al., 2002). In this study, four indels recurred at positions c.3637, c.4379, and c.4825 in three, three, and two patients, respectively, all the indels are within the poly-A runs. Approximately 50% of the nonsense variants (7/18) occurred at arginine codons (Table 5). These seven nonsense variants involved a change from the CGA for arginine to the TGA, a premature termination codon, which is consistent with earlier reports that arginine is a hotspot for nonsense variants. (McGinniss et al., 1993; Xue et al., 2010). It is commonly believed that patients with protein-truncating variants predicted to produce new stop codons develop severe diseases. However, the two patients (HA 5-6) with such variants in this study were only moderately affected. HA5, who harbored a c.6496C>T (p.Arg2166*) variant, was a one-year-old boy who underwent FVIII:C testing after experiencing bruising and swelling around the eye following a fall. The test revealed an FVIII:C level of 1.2%. Interestingly, another patient with HA and the same c.6496C>T (p.Arg2166*) variant was severely affected (Table 4). This difference highlights the phenotypic variability associated with *F8* variants. The mother of HA6 was referred to our clinic for prenatal diagnosis, and her affected son had an FVIII:C level of 2%. Unfortunately, these two patients were not available for follow-up. Although the two variants are expected to result in a null phenotype, functional assays are required to confirm their effects, as some protein-truncating variants may display trace or residual expression and/or functional levels. Similarly, patients with certain protein-truncating variants are moderately or mildly affected (Miller et al., 2012; Testa et al., 2023).

**TABLE 5:** Detailed description of variants in *F8* identified in this cohort.

Variant type	Nucleotide	Amino acid	Exon	Domain^a^	AutoPVS1^b^	REVEL^c^	Novel/Known^d^	FVIII:C (%)^e^	Criteria^f^	Classification^g^	Patient number	Origin	Severity	Inhibitor (BU/mL)
Missense	c.201G>C	p.Lys67Asn	2	A1	-	0.845	Known	1.5	PS4, PM1, PM2-PP, PP3-M, PP4	P	1	NA	Moderate	NA
	c.262A>G	p.Met88Val	2	A1	-	0.783	Known	<1	PS4, PM1, PM2-PP, PP3-M, PP4	P	1	M	Severe	0
	c.389G>A	p.Gly130Glu	3	A1	-	0.862	Known	<1	PS4-PP, PM1, PM2-PP, PP3-M, PP4	LP	1	M	Severe	0
	c.394G>A^#^	p.Glu132Lys	4	A1	-	0.609	Novel	<1	BP2, BP4	LB	1	NA	Severe	NA
	c.536C>G	p.Ser179Cys	4	A1	-	0.946	Known	0.1	PS4-PP, PM1, PM2-PP, PP3-M, PP4	LP	1	M	Severe	0
	c.608T>C	p.Leu203Pro	5	A1	-	0.848	Known	<1	PS4-M, PM1, PM2-PP, PP3-M, PP4	LP	1	NA	Severe	0
	c.871G>A^&^	p.Glu291Lys	7	A1	-	0.828	Known	4.7/5	PS4, PM1, PM2-PP, PP3-M, PP4	P	2	M/NA	Moderate/Moderate	0/0
	c.976C>G	p.Leu326Val	7	A1	-	0.536	Novel	24.5	PM1, PM2-PP, PP4	VUS	1	M	Mild	NA
	c.1420G>A	p.Gly474Arg	9	A2	-	0.972	Known	<1	PS4-PP, PM1, PM2-PP, PP3-M, PP4	LP	1	M	Severe	0
	c.1505T>G	p.Val502Gly	10	A2	-	0.879	Known	2	PS4-M, PM1, PM2-PP, PP3-M, PP4	LP	1	M	Moderate	0
	c.1649G>A	p.Arg550His	11	A2	-	0.738	Known	19.1	PS4, PM1, PM2-PP, PP3, PP4	LP	1	NA	Mild	0
	c.1750C>A	p.Gln584Lys	11	A2	-	0.909	Known	1.7	PS4-M, PM1, PM2-PP, PP3-M, PP4	LP	1	NA	Moderate	NA
	c.1764C>G	p.Asp588Glu	12	A2	-	0.825	Known	7/13	PS4-PP, PM1, PM2-PP, PP3-M, PP4	LP	2	M/M	Mild/Mild	0/0
	c.1796A>G	p.Asp599Gly	12	A2	-	0.964	Known	0.9	PS4-PP, PM1, PM2-PP, PP3-M, PP4	LP	1	M	Severe	0
	c.1808G>T	p.Ser603Ile	12	A2	-	0.971	Known	0.8	PS4-PP, PM1, PM2-PP, PP3-M, PP4	LP	1	M	Severe	0
	c.1834C>T	p.Arg612Cys	12	A2	-	0.808	Known	9/9	PM1, PM2-PP, PP3-M, PP4, BS2	VUS	2	M/M	Mild/Mild	0/0
	c.1891A>T	p.Asn631Tyr	12	A2	-	0.859	Novel	0.8	PM1, PM2-PP, PP3-M, PP4	LP	1	M	Severe	0
	c.1913G>A	p.Gly638Asp	13	A2	-	0.970	Known	0.8	PS4-M, PM1, PM2-PP, PP3-M, PP4	LP	1	M	Severe	0
	c.2003T>A	p. Leu668His	13	A2	-	0.736	Known	NA	PS4-PP, PM1, PM2-PP, PP3	VUS	1	M	?	NA
	c.2048A>G	p.Tyr683Cys	13	A2	-	0.696	Known	0.1/NA	PS4, PM1, PM2-PP, PP3, PP4	LP	2	NA/NA	Severe/?	0/NA
	c.2097G>A	p.Met699Ile	13	A2	-	0.901	Known	NA	PS4-PP, PM1, PM2-PP, PP3-M	LP	1	NA	?	NA
	c.2099C>G	p.Ser700Trp	13	A2	-	0.619	Novel	4.9	PM1, PM2-PP, PP4	VUS	1	NA	Moderate	0
	c.2535C>A^&^	p.Asp845Glu	14	B	-	0.242	Known	4.7	BS2, BP2, BP4	LB	1	NA	Moderate	NA
	c.5246T>C	p.Phe1749Ser	15	A3	-	0.869	Known	0.9	PS4-M, PM1, PM2-PP, PP3-M, PP4	LP	1	NA	Severe	0
	c.5399G>A	p.Arg1800His	16	A3	-	0.960	Known	2/1.8	PS4, PM1, PM2-PP, PP3-M, PP4	LP	2	NA/NA	Moderate/Moderate	0/0
	c.5707G>A	p.Asp1903Asn	17	A3	-	0.877	Known	3	PS4-M, PM1, PM2-PP, PP3-M, PP4	LP	1	NA	Moderate	0
	c.6103G>A	p.Val2035Met	19	A3	-	0.936	Known	2	PS4, PM1, PM2-PP, PP3-M, PP4	P	1	M	Moderate	0
	c.6113A>G	p.Asn2038Ser	19	A3	-	0.254	Known	8	PS4-M, PM1, PM2-PP, PP4	LP	1	NA	Mild	0
	c.6200C>T	p.Pro2067Leu	21	C1	-	0.979	Known	<1	PS4-M, PM1, PM2-PP, PP3-M, PP4	LP	1	M	Severe	0
	c.6361A>T	p.Ile2121Phe	22	C1	-	0.584	Novel	13	PM1, PM2-PP, PP4	VUS	1	M	Mild	NA
	c.6532C>T	p.Arg2178Cys	23	C1	-	0.875	Known	12.9	PS4, PM1, PM2-PP, PP3-M, PP4	P	1	M	Mild	0
	c.6545G>A	p.Arg2182His	23	C1	-	0.964	Known	2	PS4, PM1, PM2-PP, PP3-M, PP4	P	1	M	Moderate	0
	c.6977G>A	p.Arg2326Gln	25	C2	-	0.836	Known	NA	PS4, PM1, PM2-PP, PP3-M	LP	1	NA	?	NA
	c.6977G>T	p.Arg2326Leu	25	C2	-	0.882	Known	<1	PS4, PM1, PM2-PP, PP3-M, PP4	P	1	M	Severe	0
	c.6996G>C	p.Trp2332Cys	26	C2	-	0.877	Known	6/16	PS4-PP, PM1, PM2-PP, PP3-M, PP4	LP	2	M/NA	Mild/Mild	0/0
	c.7021G>C	p.Glu2341Gln	26	C2	-	0.783	Known	1.5	PS4-PP, PM1, PM2-PP, PP3-M, PP4	LP	1	M	Moderate	0
Nonsense	c.209_212del	p.Phe70*	2	A1	Very Strong	-	Known	<1	PVS1, PS4, PM2-PP, PP4	P	1	M	Severe	2.5
	c.1063C>T	p.Arg355*	8	-	Very Strong	-	Known	0.7	PVS1, PS4, PM2-PP, PP4	P	1	M	Severe	NA
	c.1290T>A	p.Tyr430*	9	A2	Very Strong	-	Novel	<1	PVS1, PM2-PP, PP4	P	1	M	Severe	0
	c.1726G>T	p.Glu576*	11	A2	Very Strong	-	Known	<1	PVS1, PS4-M, PM2-PP, PP4	P	1	M	Severe	0
	c.1804C>T	p.Arg602*	12	A2	Very Strong	-	Known	0.1/<1	PVS1, PS4, PM2-PP, PP4	P	2	M/M	Severe/Severe	0/0
	c.1967G>A	p.Trp656*	13	A2	Very Strong	-	Known	0	PVS1, PS4-M, PM2-PP, PP4	P	1	M	Severe	0
	c.2440C>T	p.Arg814*	14	B	Very Strong	-	Known	<1/NA	PVS1, PS4, PM2-PP, PP4	P	2	M/NA	Severe/?	0/NA
	c.2891C>A	p.Ser964*	14	B	Very Strong	-	Novel	<1	PVS1, PM2-PP, PP4	P	1	M	Severe	0
	c.4770T>A	p.Tyr1590*	14	B	Very Strong	-	Known	0.1/0.9	PVS1, PS4-M, PM2-PP, PP4	P	2	NA/NA	Severe/Severe	0/8
	c.4804C>T	p.Gln1602*	14	B	Very Strong	-	Known	<1	PVS1, PS4-PP, PM2-PP, PP4	P	1	M	Severe	0
	c.5063C>A	p.Ser1688*	14	-	Very Strong	-	Novel	NA	PVS1, PS4-PP, PM2-PP	P	1	M	?	NA
	c.5143C>T	p.Arg1715*	14	A3	Very Strong	-	Known	0.1/0.1/<1	PVS1, PS4, PM2-PP, PP4	P	3	M/M/NA	Severe/Severe/Severe	0/0/NA
	c.5882G>A	p.Trp 1961*	18	A3	Very Strong	-	Known	0	PVS1, PS4-M, PM2-PP, PP4	P	1	NA	Severe	0
	c.5953C>T	p.Arg 1985*	18	A3	Very Strong	-	Known	NA	PVS1, PS4, PM2-PP	P	1	M	?	NA
	c.6037G>T	p. Gly 2013*	19	A3	Very Strong	-	Novel	0.6	PVS1, PM2-PP, PP4	P	1	NA	Severe	0
	c.6393G>A	p.Trp2131*	22	C1	Very Strong	-	Known	0.8	PVS1, PS4-M, PM2-PP, PP4	P	1	M	Severe	28.8
	c.6403C>T	p.Arg2135*	22	C1	Very Strong	-	Known	0.1	PVS1, PS4, PM2-PP, PP4	P	1	NA	Severe	0
	c.6496C>T	p.Arg2166*	23	C1	Very Strong	-	Known	1.2/0.9	PVS1, PS4, PM2-PP, PP4	P	2	NA/NA	Moderate/Severe	0/6.4
Indel	c.795dup	p.(Gly266Trpfs*19)	7	A1	Very Strong	-	Novel	<1	PVS1, PM2-PP, PP4	P	1	NA	Severe	NA
	c.1427del	p.Gly477Glufs*5	9	A2	Very Strong	-	Novel	<1	PVS1, PM2-PP, PP4	P	1	NA	Severe	20
	c.1454del	p.Lys485Argfs*30	10	A2	Very Strong	-	Novel	<1	PVS1, PM2-PP, PP4	P	1	M	Severe	0
	c.1959_1963dup	p.Tyr655Trpfs*7	13	A2	Very Strong	-	Novel	<1	PVS1, PM2-PP, PP4	P	1	M	Severe	0
	c.2354_2355del	p.Ile785Argfs*4	14	B	Very Strong	-	Known	<1	PVS1, PS4-M, PM2-PP, PP4	P	1	M	Severe	0
	c.2945dup	p.Asn982Lysfs*9	14	B	Very Strong	-	Known	<1	PVS1, PS4, PM2-PP, PP4	P	1	M	Severe	0
	c.3235dup	p.(Arg1079Lysfs*8)	14	B	Very Strong	-	Novel	NA	PVS1, PM2-PP	LP	1	NA	?	NA
	c.3348del	p.(Phe1116Leufs*22)	14	B	Very Strong	-	Novel	NA	PVS1, PM2-PP	LP	1	NA	?	NA
	c.3637dup	p.Ile1213Asnfs*28	14	B	Very Strong	-	Known	<1	PVS1, PS4, PM2-PP, PP4	P	1	NA	Severe	0
	c.3637del	p.Ile1213Phefs*5	14	B	Very Strong	-	Known	<1/<1	PVS1,PS4, PM2-PP, PP4	P	2	M/M	Severe/Severe	0/0
	c.3851_3852del	p.Thr1284Serfs*35	14	B	Very Strong	-	Known	<1	PVS1, PS4-PP, PM2-PP, PP4	P	1	M	Severe	0
	c.4052_4053ins10	p.Ile1351Metfs*7	14	B	Very Strong	-	Novel	<1	PVS1, PM2-PP, PP4	P	1	M	Severe	0
	c.4121_4124del	p.Ile1374Thrfs*49	14	B	Very Strong	-	Known	<1	PVS1, PS4-PP, PM2-PP, PP4	P	1	M	Severe	0
	c.4342_4343ins	p.Gln1448Leufs*31	14	B	Very Strong	-	Novel	<1	PVS1, PM2-PP, PP4	P	1	M	Severe	0
	c.4379del	p.Asn1460Ilefs*5	14	B	Very Strong	-	Known	<1	PVS1, PS4, PM2-PP, PP4	P	1	M	Severe	0
	c.4379dup	p.Asn1460Lysfs*2	14	B	Very Strong	-	Known	<1/<1	PVS1, PS4, PM2-PP, PP4	P	2	M/M	Severe/Severe	0/0
	c.4825dup	p.Thr1609Asnfs*4	14	B	Very Strong	-	Known	<1/<1	PVS1, PS4, PM2-PP, PP4	P	2	M/*de novo*	Severe/Severe	0/0
	c.5174del	p.Leu1725Profs*6	14	A3	Very Strong	-	Novel	<1	PVS1, PM2-PP, PP4	P	1	M	Severe	0
	c.5818_5820delinsCACAT	p.Ile1940Hisfs*6	18	A3	Very Strong	-	Novel	<1	PVS1, PM2-PP, PP4	P	1	M	Severe	33.6
	c.5963_5964del	p.(Glu1988Glyfs*3)	18	A3	Very Strong	-	Known	2	PVS1, PS4-PP, PM2-PP, PP4	P	1	M	Moderate	NA
	c.6212_6225del	p.Leu2072Ilefs*49	21	C1	Very Strong	-	Novel	<1	PVS1, PM2-PP, PP4	P	1	M	Severe	8.4
	c.6988dup	p.Gln2330Profs*55	26	C2	Strong	-	Known	<1	PVS1-PS, PS4-PP, PM2-PP, PP4	LP	1	NA	Severe	0
Canonical splice site	c.1443 + 1G>A	-	-	-	Very Strong	-	Known	<1	PVS1, PS4-M, PM2-PP, PP4	P	1	NA	Severe	0
	c.5815 + 1G>A	-	-	-	Very Strong	-	Known	<1	PVS1, PS4-M, PM2-PP, PP4	P	1	M	Severe	5.2
Gross deletion	Exon 1 deletion^#^	-	1	-	-	-	Known	<1	PVS1, PS4-VS, PM2-PP, PP4	P	1	M	Severe	0
	Exon 5–6 deletion	-	5-6	-	-	-	Known	<1	PVS1, PS4, PM2-PP, PP4	P	1	NA	Severe	0
	Exon 14 deletion	-	14	-	-	-	Known	<1	PVS1, PS4, PM2-PP, PP4	P	1	NA	Severe	25.6
	Exon 15–26 deletion	-	15-26	-	-	-	Known	<1	PVS1, PS4-M, PM2-PP, PP4	P	1	NA	Severe	25.6
	Exon 26 deletion	-	26	-	-	-	Known	<1/<1	PVS1-S, PS4-VS, PM2-PP, PP4	P	2	M/NA	Severe/Severe	0/0

^a^When a variant located at a domain of F8 except for the B domain, the moderate evidence of pathogenicity (PM1) can be applied. ^b^The prediction result of “Very Strong” by autoPVS1 indicated that a very strong evidence pathogenicity (PVS1) can be applied, and “strong” indicated that the strength should be reduced. For the one or muti exon(s) deletion, the classification was according to the recommendation by Abou Tayoun et al. (2018). ^c^The thresholds for the levels of evidence for pathogenicity (PP3) were: ≥0.733 for moderate, [0.644, 0.733) for PP3, and the level of evidence for benign (BP4) were: ≤0.003 for very strong, (0.003, 0.016] for strong, (0.016, 0.183] for moderate and (0.183, 0.290] for BP4. ^d^The criteria for pathogenicity “PS4_Supporting”, “PS4_Moderate”, “PS4”, and “PS4_Very Strong” can be applied when 1, 2, 4, and 16 cases respectively had been reported. ^e^FVIII:C indicated the plasma factor VIII, clotting activity, it is shown as % of normal. The supporting evidence for pathogenicity (PP4) can be applied when the FVIII:C is below 40%. ^f^This column listed the criteria applied for the variant. ^g^The classification of variant is based on the applied criteria according to the scoring rules in the ACMG/AMP, recommendations (Richards et al., 2015). LP: Likely Pathogenic. M: Mother. NA: Not available. P: Pathogenic. VUS: Variant of uncertain significance.^&^This two variants co-occurred in one patient. ^#^This two variants co-occurred in one patient.

#### Canonical splice site variants

Two canonical splice site variants were identified in this cohort, accounting for 1.3% of all patients and 1.7% of the cases classified as severe (Table 1). Canonical splice site variants were the least represented variant type in this study, which may partially result from the limited number of cases.

#### Missense variants

Our analysis identified 36 distinct missense variants across the *F8* gene in 40 patients, with an exception for exons 1, 6, 8, 18, 20, and 24. We found a c.394G>A (p.Glu132Lys) variant along with the exon 1 deletion in one patient; this variant, located in the A1 domain, is absent from the gnomAD database and previously unreported in HA patients (https://f8-db.eahad.org/). The variant c.2535C>A (p.Asp845Glu) was discovered alongside the known variant c.871G>A (p.Glu291Lys) in one patient. This variant has been implicated in HA patients, and *in vitro* analyses for this variant did not demonstrate a significant reduction or revealed only a mild decrease in FVIII:C (Zhang et al., 1999; Ogata et al., 2011; Pahl et al., 2014). Notably, this variant is located in exon 14, the region coding for the B domain of FVIII. The B domain is partially spliced off the mature protein and lacks procoagulant activity (Thompson, 2003; Camire and Bos, 2009); it is responsible for FVIII processing and trafficking via its N-linked oligosaccharides (Ogata et al., 2011; Testa et al., 2023). It is well known that variants of the B domain are unlikely to cause severe HA and rarely cause mild HA (Ogata et al., 2011). One explanation could be that the observed variant(s) in the B domain may have a minor impact on FVIII intracellular processing. Regardless of the two likely benign variants, c.394G>A (p.Glu132Lys) and c.2535C>A (p.Asp845Glu), all the missense variants in this study were located outside the B domain.

#### Large deletion

MLPA identified five large deletions within the *F8* gene among six patients, with two deletions extending over multiple exons—specifically from exon 5 to 6 and exon 15 to 26. To identify the breaking point of the gross deletion of exon 26, multiple primers were designed around exon 26 to amplify the sequences, and an approximately 1 kb PCR product was amplified with the primers (forward: TGT​AAT​TCA​GTT​AGT​CAC​AGG​AT, reverse: TAA​AAC​AAC​CTA​TTC​ACT​ACC​AC). Sequencing of the PCR product indicated the breakpoint at positions chrX:154083181 upstream and chrX:154053438 downstream of exon 26, resulting in a 29,742 bp deletion. The same deletion was confirmed in the proband’s mother, suggesting hereditary transmission. While these exon deletions have been previously reported, this is the first report to delineate the breakpoint for the exon 26 deletion.

### Variants in *F9*

In the *F9* gene, 24 unique variants were observed in 30 of 32 male patients with FIX:C levels <40%. These included 16 missense variants, two nonsense variants, one small deletion, two gross deletions, one canonical splice site variant and two noncanonical splice site variants (Table 1). Missense variants were the most prominent variant type in all severities and were distributed across all exons, except exon 1 and 3. The majority of missense variants (9/16) occurred in exons 7 and 8 (Table 6), which encode the serine protease (SP) domain. The c.571C>T (p.Arg191Cys) variant recurred in four patients (4/30) (Table 6), which is consistent with the high frequency described in a previous report (Branchini et al., 2022), and more than 100 HB patients with variants at p. Arg191 have been reported; there are two variants at p. Arg43 (c.127C>T (p.Arg43Trp) and c.128G>A (p.Arg43Gln)) (Table 6) in this study, but more than 100 HB patients with variants at p. Arg43 have been reported in the EAHAD *F9* databases. The prevalent variants observed at positions p. Arg43 and p. Arg191 suggest mutational hotspots, possibly resulting from the inherently mutagenic nature of CpG dinucleotides at these positions (Morgan et al., 1995). All the nonsense variants, small deletion, gross deletions and canonical splice site variant were predicted to cause protein truncation. The two noncanonical splice site variants were predicted to affect the splicing of the exon in *F9*. No *F9* variants were identified in the two patients with FIX:C levels below 40%, which could be due to naturally low FIX:C levels in these patients, as they were under the age of 6 months.

**TABLE 6:** Detailed description of variants in *F9* identified in this cohort.

Variant type	Nucleotide	Amino acid	Exon	Domain^a^	AutoPVS1^b^	Splice AI^c^	REVEL^d^	Novel/Known^e^	FIX:C (%)^f^	Criteria^g^	Classification^h^	Patient number	Origin	Severity	Inhibitor (BU/mL)
Missense	c.127C>T	p.Arg43Trp	2	Gla	-	-	0.692	Known	2.3/1.7	PS4-VS, PM1, PM2-PP, PP3, PP4	P	2	NA/NA	Moderate/Moderate	0/0
	c.128G>A	p.Arg43Gln	2	Gla	-	-	0.710	Known	<1	PS4-VS, PM1, PM2-PP, PP3, PP4	P	1	NA	Severe	0
	c.316G>A	p.Gly106Ser	4	EGF1	-	-	0.905	Known	8	PM1, PM2-PP, PP3-M, PP4, BS2	VUS	1	NA	Mild	0
	c.370G>A	p.Glu124Lys	4	EGF1	-	-	0.763	Known	3.2	PS4-M, PM1, PM2-PP, PP3, PP4	LP	1	NA	Moderate	0
	c.509G>A	p.Cys170Tyr	5	EGF2	-	-	0.971	Known	<1	PS4-PP, PM1, PM2-PP, PP3-M, PP4	LP	1	NA	Severe	0
	c.571C>T	p.Arg191Cys	6	Linker	-	-	0.808	Known	<1 in the four	PS4, PM1, PM2-PP, PP3-M, PP4	P	4	NA in the four	Severe in the four	0 in the four
	c.754T>A	p.Cys252Ser	7	SP	-	-	0.978	Novel	NA	PM1, PM2-PP, PP3-M	VUS	1	M	?	NA
	c.781T>C	p.Trp261Arg	7	SP	-	-	0.910	Known	0.9	PS4-M, PM1, PM2-PP, PP3-M, PP4	LP	1	NA	Severe	NA
	c.934T>G	p.Tyr312Asp	8	SP	-	-	0.840	Known	<1	PS4-PP, PM1, PM2-PP, PP3-M, PP4	LP	1	NA	Severe	0
	c.992T>A	p.Val331Asp	8	SP	-	-	0.816	Novel	1.8	PM1, PM2-PP, PP3-M, PP4	LP	1	NA	Moderate	NA
	c.1115T>A	p.Leu372His	8	SP	-	-	0.837	Novel	1.9/3	PM1, PM2-PP, PP3-PM, PP4	LP	2	NA/NA	Moderate/Moderate	0/0
	c.1187G>A	p.Cys396Tyr	8	SP	-	-	0.959	Known	<1	PS4-M, PM1, PM2-PP, PP3-M, PP4	LP	1	NA	Severe	0
	c.1220G>A	p.Cys407Tyr	8	SP	-	-	0.949	Known	0.3	PS4-PP, PM1, PM2-PP, PP3-M, PP4	LP	1	M	Severe	0
	c.1295G>T	p.Gly432Val	8	SP	-	-	0.933	Known	<1	PS4, PM1, PM2-PP, PP3-M, PP4	P	1	M	Severe	0
	c.1328T>C	p.Ile443Thr	8	SP	-	-	0.905	Known	2.2	PS4-M, PM1, PM2-PP, PP3-M, PP4	LP	1	NA	Moderate	0
	c.1346G>A	p.Arg449Gln	8	Signal peptide	-	-	0.588	Known	17.7	PM1, PM2-PP, PP3, PP4, BS2	VUS	1	NA	Mild	NA
Nonsense	c.223C>T	p.Arg75*	2	Gla	Very Strong	-	-	Known	0.1	PVS1, PS4, PM2-PP, PP4	P	1	NA	Severe	0.6
	c.880C>T	p.Arg294*	7	SP	Strong	-	-	Known	<1/<1	PVS1-S, PS4, PM2-PP, PP4	P	2	NA/NA	Severe/Severe	0/0
Small deletion	c.252delA	p.Thr85Leufs*19	2	Gla	Very Strong	-	-	Novel	0.1	PVS1, PM2-PP, PP4	P	1	NA	Severe	0
Gross deletion	Exon 6–7 deletion	?	6-7	-	-	-	-	Novel	<1	PVS1, PM2-PP, PP4	P	1	NA	Severe	0
	Exon 7–8 deletion	?	7-8	-	-	-	-	Known	<1	PVS1, PS4, PM2-PP, PP4	P	1	NA	Severe	1.3
Noncanonical splice site	c.252 + 3_c.252+6delGAGT	?	-	-	-	0.73	-	Known	<1	PM2-PP, PS4-M, PP3, PP4	VUS	1	NA	Severe	NA
	c.253-6T>G	?	-	-	-	0.36	-	Novel	6	PM2-PP, PP3, PP4	VUS	1	M	Mild	0
Canonical splice site	c.723 + 1G>T	?	-	-	Very Strong	-	-	Known	<1	PVS1, PS4-M, PM2-PP, PP4	P	1	NA	Severe	0

^a^When a variant located at a domain of F9, the moderate evidence of pathogenicity (PM1) can be applied. ^b^The prediction result of “Very Strong” by autoPVS1 indicated that a very strong evidence pathogenicity (PVS1) can be applied, and “strong” indicated that the strength should be reduced. For the one or muti exon(s) deletion, the classification was according to the recommendation by Abou Tayoun et al. (2018). ^c^For the splice region variant, the supporting evidence for pathogenicity (PP3) can be applied when the SpliceAI scores is >0.2. ^d^The thresholds for the levels of evidence for pathogenicity (PP3) were: ≥0.733 for moderate, [0.644, 0.733) for PP3, and the level of evidence for benign (BP4) were: ≤0.003 for very strong, (0.003, 0.016] for strong, (0.016, 0.183] for moderate and (0.183, 0.290] for BP4. ^e^The criteria for pathogenicity “PS4_Supporting”, “PS4_Moderate”, “PS4”, and “PS4_Very Strong” can be applied when 1, 2, 4, and 16 cases respectively had been reported. ^f^FIX:C indicated the plasma factor IX, clotting activity, it is shown as % of normal. The supporting evidence for pathogenicity (PP4) can be applied when the FVIII:C is below 40%. ^g^This column listed the criteria applied for the variant. ^h^The classification of variant is based on the applied criteria according to the scoring rules in the ACMG/AMP, recommendations (Richards et al., 2015). EGF: Epidermal growth factor. LP: Likely Pathogenic. M: Mother. NA: Not available. P: Pathogenic. SP: serine protease. VUS: variant of uncertain significance.

### Novel *F8* and *F9* variants

Twenty novel variants were identified among the 83 *F8* variants detected. These included 11 indels, five missense variants, and four nonsense variants (Table 1), representing a novel variant detection rate of 24%. The low yield of novel variants likely reflects the extensive genetic testing for this disease, particularly in the era of whole-exome sequencing. It remains to be seen which fraction of variants in Chinese patients may be genuinely distinct from those in other ethnic groups. None of the novel variants had been cataloged in the gnomAD and *F8* EAHAD databases. Except for c.394G>A (p.Glu132Lys), the other four missense variants, c.976C>G (p.Leu326Val), c.1891A>T (p.Asn631Tyr), c.2099C>G (p.Ser700Trp), and c.6361A>T (p.Ile2121Phe), occurred at conserved sites relative to those in other species (data not shown), affecting the A1, A2, and C1 domains. The remaining 15 novel indel and nonsense variants were predicted introduce premature stop codons and result in C-terminal truncation of the protein (Table 5).

In the *F9* gene, six novel variants were identified among the 24 *F9* variants detected, including three missense variants, one noncanonical splice site variant, one small deletion, and one large deletion (Table 1). None of these variants were recorded in the gnomAD and *F9* EAHAD databases. Missense variants c.754T>A (p.Cys252Ser), c.992T>A (p.Val331Asp), and c.1115T>A (p.Leu372His) were clustered in the SP domain, and the mutant amino acids were also conserved among species. The variant c.754T>A (p.Cys252Ser) has a novel nucleotide change, but a variant c.755G>C (p.Cys252Ser) with the identical amino acid alteration has been reported in the EAHAD *F9* database. Only one novel small deletion, c.252del (p.Thr85Leufs*19), was detected. It resulted in a premature stop codon, causing a truncation of 357 amino acids at the C-terminus of the coding protein. In addition, we identified a deletion in exons 6 and 7 via MLPA. The noncanonical splice site variant c.253-6T>G was the only novel variant of uncertain significance (Table 6).

## Conclusion

In conclusion, this analysis expands the variant spectrum of *F8* and *F9* in the southern Chinese population, identifying 26 novel variants and providing insights into the clinical features and prenatal diagnosis of hemophilia. This study contributes to our understanding of the genetic basis of the disease in this population and emphasizes that the effects of *F8*/*F9* variants on FVIII/FIX protein levels and function should be thoroughly analyzed.

## Data Availability

The raw data supporting the conclusion of this article will be made available by the authors, without undue reservation.

## Abbreviations

APTT, Activated partial thromboplastin time; ACMG, American College of Medical Genetics; AutoPVS1, Automatic PVS1 interpretation; A, Stand-alone; B, Benign; BU, Bethesda unit; EAHAD, European Association for Haemophilia and Allied Disorders; FVIII, Coagulation factor VIII; FVIII: C, Factor VIII clotting activity; FIX, Coagulation factor IX; FIX: C, Factor IX clotting activity; gnomAD, Genome Aggregation Database; HA, Hemophilia A; HB, Hemophilia B; Indel, Small deletion and insertion; IVS 1, Intron 1; IVS 22, Intron 22; LB, Likely benign; LP, Likely pathogenic; MLPA, Multiplex Ligation Dependent Probe Amplification; M, Moderate; NMD, Nonsense-medicated decay; PCR, Polymerase Chain Reaction; PP, Supporting; P, pathogenic; REVEL, Rare Exome Variant Ensembl Learner; SP, Serine protease; S, Strong; VS, Very strong; VUS, Variant of uncertain significance.

## References

[B1] Abelleyro M. M. Radic C. P. Marchione V. D. Waisman K. Tetzlaff T. Neme D. (2020). Molecular insights into the mechanism of nonrecurrent F8 structural variants: full breakpoint characterization and bioinformatics of DNA elements implicated in the upmost severe phenotype in hemophilia A. Hum. Mutat. 41, 825–836. 10.1002/humu.23977 31898853

[B2] Abou Tayoun A. N. Pesaran T. DiStefano M. T. Oza A. Rehm H. L. Biesecker L. G. (2018). Recommendations for interpreting the loss of function PVS1 ACMG/AMP variant criterion. Hum. Mutat. 39, 1517–1524. 10.1002/humu.23626 30192042 PMC6185798

[B3] Aledort L. M. Dimichele D. M. (1998). Inhibitors occur more frequently in African-American and Latino haemophiliacs. Haemophilia 4, 68. 10.1046/j.1365-2516.1998.0146c.x 9873872

[B4] Andrew M. Paes B. Milner R. Johnston M. Mitchell L. Tollefsen D. M. (1987). Development of the human coagulation system in the full-term infant. Blood 70, 165–172. 10.1182/blood.v70.1.165.165 3593964

[B5] Antonarakis S. E. Rossiter J. P. Young M. Horst J. de Moerloose P. Sommer S. S. (1995). Factor VIII gene inversions in severe hemophilia A: results of an international consortium study. Blood 86, 2206–2212. 10.1182/blood.v86.6.2206.bloodjournal8662206 7662970

[B6] Bach J. E. Oldenburg J. Müller C. R. Rost S. (2016). Mutational spectrum and deep intronic variants in the factor VIII gene of haemophilia A patients. Identification by next generation sequencing. Hamostaseologie 36, S25–S28. 27824209

[B7] Bidichandani S. I. Lanyon W. G. Connor J. M. (1994). Characterisation of a 5-bp deletion in exon 4 of the factor VIII gene: concordance with slipped-mispairing at DNA replication. Hum. Genet. 94, 447–449. 10.1007/BF00201612 7927348

[B8] Bogdanova N. Markoff A. Pollmann H. Nowak-Göttl U. Eisert R. Dworniczak B. (2002). Prevalence of small rearrangements in the factor VIII gene F8C among patients with severe hemophilia A. Hum. Mutat. 20, 236–237. 10.1002/humu.9062 12204009

[B9] Bolton-Maggs P. H. Pasi K. J. (2003). Haemophilias A and B. Lancet 24, 1801–1809. 10.1016/S0140-6736(03)13405-8 12781551

[B10] Branchini A. Morfini M. Lunghi B. Belvini D. Radossi P. Bury L. (2022). F9 missense mutations impairing factor IX activation are associated with pleiotropic plasma phenotypes. J. Thromb. Haemost. 20, 69–81. 10.1111/jth.15552 34626083 PMC9298354

[B11] Camire R. M. Bos M. H. (2009). The molecular basis of factor V and VIII procofactor activation. J. Thromb. Haemost. 7, 1951–1961. 10.1111/j.1538-7836.2009.03622.x 19765210 PMC2993324

[B12] Carpenter S. L. Michael Soucie J. Sterner S. Presley R. Hemophilia Treatment Center Network HTCN Investigators (2012). Increased prevalence of inhibitors in Hispanic patients with severe haemophilia A enrolled in the Universal Data Collection database. Haemophilia 18, e260–e265. 10.1111/j.1365-2516.2011.02739.x 22250850

[B13] Castaman G. Giacomelli S. H. Mancuso M. E. D'Andrea G. Santacroce R. Sanna S. (2011). Deep intronic variations may cause mild hemophilia A. J. Thromb. Haemost. 9, 1541–1548. 10.1111/j.1538-7836.2011.04408.x 21689372

[B14] Castaman G. Matino D. (2019). Hemophilia A and B: molecular and clinical similarities and differences. Haematologica 104, 1702–1709. 10.3324/haematol.2019.221093 31399527 PMC6717582

[B15] Chang C. Y. Perng C. L. Cheng S. N. Hu S. H. Wu T. Y. Lin S. Y. (2019). Deep intronic variant c.5999-277G>A of F8 gene may be a hot spot mutation for mild hemophilia A patients without mutation in exonic DNA. Eur. J. Haematol. 103, 47–55. 10.1111/ejh.13242 31063249

[B16] Chen J. Li Q. Lin S. Li F. Huang L. Jin W. (2021). The spectrum of FVIII gene variants detected by next generation sequencing in 236 Chinese non-inversion hemophilia A pedigrees. Thromb. Res. 202, 8–13. 10.1016/j.thromres.2021.02.027 33706050

[B17] den Dunnen J. T. Dalgleish R. Maglott D. R. Hart R. K. Greenblatt M. S. McGowan-Jordan J. (2016). HGVS recommendations for the description of sequence variants: 2016 update. Hum. Mutat. 37, 564–569. 10.1002/humu.22981 26931183

[B18] Dericquebourg A. Jourdy Y. Fretigny M. Lienhart A. Claeyssens S. Ternisien C. (2020). Identification of new F8 deep intronic variations in patients with haemophilia A. Haemophilia 26, 847–854. 10.1111/hae.14134 32812322

[B19] Duncan E. Collecutt M. Street A. (2013). Nijmegen-Bethesda assay to measure factor VIII inhibitors. Methods Mol. Biol. 992, 321–333. 10.1007/978-1-62703-339-8_24 23546724

[B20] Eckhardt C. L. van Velzen A. S. Peters M. Astermark J. Brons P. P. Castaman G. (2013). Factor VIII gene (F8) mutation and risk of inhibitor development in nonsevere hemophilia A. Blood 122, 1954–1962. 10.1182/blood-2013-02-483263 23926300

[B21] Fogarty P. F. Kessler C. M. (2013). “Hemophilia A and B,” in Consultative hemostasis and thrombosis. Editors Kitchens C. S. Kessler C. M. Konkle B. A. 3 ed (Philadelphia, PA: Elsevier Saunders), 45–59.

[B23] Gouw S. C. van den Berg H. M. Oldenburg J. Astermark J. de Groot P. G. Margaglione M. (2012). F8 gene mutation type and inhibitor development in patients with severe hemophilia A: systematic review and meta-analysis. Blood 119, 2922–2934. 10.1182/blood-2011-09-379453 22282501

[B24] Guo Z. Yang L. Qin X. Liu X. Zhang Y. (2018). Spectrum of molecular defects in 216 Chinese families with hemophilia A: identification of noninversion mutation hot spots and 42 novel mutations. Clin. Appl. Thromb. Hemost. 24, 70–78. 10.1177/1076029616687848 28056528 PMC6714629

[B25] Johnsen J. M. Fletcher S. N. Dove A. McCracken H. Martin B. K. Kircher M. (2022). Results of genetic analysis of 11 341 participants enrolled in the My Life, Our Future hemophilia genotyping initiative in the United States. J. Thromb. Haemost. 20, 2022–2034. 10.1111/jth.15805 35770352

[B26] Josephson N. (2013). The hemophilias and their clinical management. Hematol. Am. Soc. Hematol. Educ. Program 2013, 261–267. 10.1182/asheducation-2013.1.261 24319189

[B27] Knobe K. E. Villoutreix B. O. Tengborn L. I. Petrini P. Ljung R. C. (2000). Factor VIII inhibitors in two families with mild haemophilia A: structural analysis of the mutations. Haemostasis 30, 268–279. 10.1159/000054143 11251334

[B28] Konkle B. A. Nakaya Fletcher S. (2000). “Hemophilia A,” in GeneReviews®. Editors Adam M. P. Everman D. B. Mirzaa G. M. Pagon R. A. Wallace S. E. Bean L. J. H. (Seattle (WA): University of Washington), 1993–2022.

[B29] Krawczak M. Cooper D. N. (1991). Gene deletions causing human genetic disease: mechanisms of mutagenesis and the role of the local DNA sequence environment. Hum. Genet. 86, 425–441. 10.1007/BF00194629 2016084

[B30] Lee K. Krempely K. Roberts M. E. Anderson M. J. Carneiro F. Chao E. (2018). Specifications of the ACMG/AMP variant curation guidelines for the analysis of germline CDH1 sequence variants. Hum. Mutat. 39, 1553–1568. 10.1002/humu.23650 30311375 PMC6188664

[B31] Lu Y. Xin Y. Dai J. Wu X. You G. Ding Q. (2018). Spectrum and origin of mutations in sporadic cases of haemophilia A in China. Haemophilia. 24, 291–298. 10.1111/hae.13402 29381227

[B32] Luna-Záizar H. González-Alcázar J. Á. Evangelista-Castro N. Aguilar-López L. B. Ruiz-Quezada S. L. Beltrán-Miranda C. P. (2018). F8 inversions of introns 22 and 1 confer a moderate risk of inhibitors in Mexican patients with severe hemophilia A. Concordance analysis and literature review. Blood Cells Mol. Dis. 71, 45–52. 10.1016/j.bcmd.2018.02.003 29544691

[B33] Male C. Andersson N. G. Rafowicz A. Liesner R. Kurnik K. Fischer K. P. (2021). Inhibitor incidence in an unselected cohort of previously untreated patients with severe haemophilia B: a PedNet study. Haematologica 106, 123–129. 10.3324/haematol.2019.239160 31919092 PMC7776246

[B34] Mannucci P. M. Franchini M. (2013). Is haemophilia B less severe than haemophilia A? Haemophilia 19, 499–502. 10.1111/hae.12133 23517072

[B35] McGinniss M. J. Kazazian H. H. Jr Hoyer L. W. Bi L. Inaba H. Antonarakis S. E. (1993). Spectrum of mutations in CRM-positive and CRM-reduced hemophilia A. Genomics 15, 392–398. 10.1006/geno.1993.1073 8449505

[B36] McVey J. H. Rallapalli P. M. Kemball-Cook G. Hampshire D. J. Giansily-Blaizot M. Gomez K. (2020). The European association for haemophilia and allied disorders (EAHAD) coagulation factor variant databases: important resources for haemostasis clinicians and researchers. Haemophilia 26, 306–313. 10.1111/hae.13947 32166871

[B37] Mester J. L. Ghosh R. Pesaran T. Huether R. Karam R. Hruska K. S. (2018). Gene-specific criteria for PTEN variant curation: recommendations from the ClinGen PTEN expert panel. Hum. Mutat. 39, 1581–1592. 10.1002/humu.23636 30311380 PMC6329583

[B38] Miller C. H. Benson J. Ellingsen D. Driggers J. Payne A. Kelly F. M. (2012). F8 and F9 mutations in US haemophilia patients: correlation with history of inhibitor and race/ethnicity. Haemophilia 18, 375–382. 10.1111/j.1365-2516.2011.02700.x 22103590

[B39] Morgan G. E. Figueiredo M. S. Winship P. R. Baker R. Bolton-Maggs P. H. Brownlee G. G. (1995). The high frequency of the -6G-->A factor IX promoter mutation is the result both of a founder effect and recurrent mutation at a CpG dinucleotide. Br. J. Haematol. 89, 672–674. 10.1111/j.1365-2141.1995.tb08388.x 7734378

[B40] Nakaya S. Liu M. L. Thompson A. R. (2001). Some factor VIII exon 14 frameshift mutations cause moderately severe haemophilia A. Br. J. Haematol. 115, 977–982. 10.1046/j.1365-2141.2001.03173.x 11843836

[B41] Ogata K. Selvaraj S. R. Miao H. Z. Pipe S. W. (2011). Most factor VIII B domain missense mutations are unlikely to be causative mutations for severe hemophilia A: implications for genotyping. Thromb. Haemost. 9, 1183–1190. 10.1111/j.1538-7836.2011.04268.x PMC3111924 21645226

[B42] Oldenburg J. Pavlova A. (2006). Genetic risk factors for inhibitors to factors VIII and IX. Haemophilia 6, 15–22. 10.1111/j.1365-2516.2006.01361.x 17123389

[B43] Orzack S. H. Stubblefield J. W. Akmaev V. R. Colls P. Munné S. Scholl T. (2015). The human sex ratio from conception to birth. Proc. Natl. Acad. Sci. U. S. A. 112, E2102–E2111. 10.1073/pnas.1416546112 25825766 PMC4413259

[B44] Pahl S. Pavlova A. Driesen J. Oldenburg J. (2014). Effect of F8 B domain gene variants on synthesis, secretion, activity and stability of factor VIII protein. Thromb. Haemost. 111, 58–66. 10.1160/TH13-01-0028 24108539

[B45] Pejaver V. Byrne A. B. Feng B. J. Pagel K. A. Mooney S. D. Karchin R. (2022). Calibration of computational tools for missense variant pathogenicity classification and ClinGen recommendations for PP3/BP4 criteria. Am. J. Hum. Genet. 109, 2163–2177. 10.1016/j.ajhg.2022.10.013 36413997 PMC9748256

[B46] Plug I. Mauser-Bunschoten E. P. Bröcker-Vriends A. H. van Amstel H. K. van der Bom J. G. van Diemen-Homan J. E. (2006). Bleeding in carriers of hemophilia. Blood 108, 52–56. 10.1182/blood-2005-09-3879 16551972

[B47] Rallapalli P. M. Kemball-Cook G. Tuddenham E. G. Gomez K. Perkins S. J. (2013). An interactive mutation database for human coagulation factor IX provides novel insights into the phenotypes and genetics of hemophilia B. J. Thromb. Haemost. 11, 1329–1340. 10.1111/jth.12276 23617593

[B48] Richards S. Aziz N. Bale S. Bick D. Das S. Gastier-Foster J. (2015). Standards and guidelines for the interpretation of sequence variants: a joint consensus recommendation of the American College of medical genetics and Genomics and the association for molecular pathology. Genet. Med. 17, 405–424. 10.1038/gim.2015.30 25741868 PMC4544753

[B49] Rosendaal F. R. Palla R. Garagiola I. Mannucci P. M. Peyvandi F. SIPPET Study Group (2017). Genetic risk stratification to reduce inhibitor development in the early treatment of hemophilia A: a SIPPET analysis. Blood 130, 1757–1759. 10.1182/blood-2017-06-791756 28768627

[B50] Testa M. F. Lombardi S. Bernardi F. Ferrarese M. Belvini D. Radossi P. (2023). Translational readthrough at F8 nonsense variants in the factor VIII B domain contributes to residual expression and lowers inhibitor association. Haematologica 108, 472–482. 10.3324/haematol.2022.281279 35924581 PMC9890017

[B51] Thompson A. R. (2003). Structure and function of the factor VIII gene and protein. Semin. Thromb. Hemost. 29, 11–22. 10.1055/s-2003-37935 12640560

[B52] Xue F. Zhang L. Sui T. Ge J. Gu D. Du W. (2010). Factor VIII gene mutations profile in 148 Chinese hemophilia A subjects. Eur. J. Haematol. 85, 264–272. 10.1111/j.1600-0609.2010.01481.x 20528906

[B53] Zhang Y. Z. Liu J. X. Shao H. Z. Chi Z. W. Wang H. L. Chen S. J. (1999). Characterization of genetic defects of hemophilia A in mainland China. Genet. Anal. 15, 205–207. 10.1016/s1050-3862(99)00005-4 10609755

